# Nutrition, Obesity, and Seborrheic Dermatitis: Systematic Review

**DOI:** 10.2196/50143

**Published:** 2024-08-05

**Authors:** Emily Woolhiser, Noah Keime, Arya Patel, Isaac Weber, Madeline Adelman, Robert P Dellavalle

**Affiliations:** 1 College of Osteopathic Medicine Kansas City University Kansas City, MO United States; 2 School of Medicine University of Colorado Aurora, CO United States; 3 School of Medicine Case Western Reserve University Cleveland, OH United States; 4 Mercy Hospital St. Louis St Louis, MO United States; 5 Department of Dermatology University of Minnesota Minneapolis, MN United States

**Keywords:** seborrheic dermatitis, systematic review, diet, nutritional supplements, alcohol, BMI, body mass index, skin, review methods, review methodology, nutrition, nutritional, supplement, supplements, dermatology, dermatitis, skin, nutrient, nutrients, micronutrient, micronutrients, vitamin, vitamins, mineral, minerals, obesity, obese, weight

## Abstract

**Background:**

Pathogenesis of seborrheic dermatitis involves lipid secretion by sebaceous glands, Malassezia colonization, and an inflammatory response with skin barrier disruption. Each of these pathways could be modulated by diet, obesity, and nutritional supplements. Current treatment options provide only temporary control of the condition; thus, it is essential to recognize modifiable lifestyle factors that may play a role in determining disease severity.

**Objective:**

This study aimed to summarize published evidence on diet, nutritional supplements, alcohol, obesity, and micronutrients in patients with seborrheic dermatitis and to provide useful insights into areas of further research.

**Methods:**

A literature search of Scopus, PubMed, and MEDLINE (Ovid interface) for English language papers published between 1993 and 2023 was conducted on April 16, 2023. Case-control studies, cohort studies, and randomized controlled trials with 5 or more subjects conducted on adult participants (>14 years) were included, case reports, case series, and review papers were excluded due to insufficient level of evidence.

**Results:**

A total of 13 studies, 8 case-control, 3 cross-sectional, and 2 randomized controlled trials, involving 13,906 patients were included. Seborrheic dermatitis was correlated with significantly increased copper, manganese, iron, calcium, and magnesium concentrations and significantly lower serum zinc and vitamin D and E concentrations. Adherence to the Western diet was associated with a higher risk for seborrheic dermatitis in female patients and an increased consumption of fruit was associated with a lower risk of seborrheic dermatitis in all patients. The prebiotic Triphala improved patient satisfaction and decreased scalp sebum levels over 8 weeks. Most studies find associations between regular alcohol use and seborrheic dermatitis, but the association between BMI and obesity on seborrheic dermatitis severity and prevalence is mixed.

**Conclusions:**

This review sheds light on specific promising areas of research that require further study, including the need for interventional studies evaluating serum zinc, vitamin D, and vitamin E supplementation for seborrheic dermatitis. The negative consequences of a Western diet, alcohol use, obesity, and the benefits of fruit consumption are well known; however, to fully understand their specific relationships to seborrheic dermatitis, further cohort or interventional studies are needed.

**Trial Registration:**

PROSPERO CRD42023417768; https://tinyurl.com/bdcta893

## Introduction

Seborrheic dermatitis is a chronic inflammatory skin disease that commonly presents on the face, scalp, and chest [1]. Seborrheic dermatitis affects approximately 5% of the global population, while its noninflammatory form affects closer to 50% of individuals [2]. Seborrheic dermatitis prefers males of all ethnicities and peaks in the first 3 months of life and again at puberty, where it then reaches an apex at 40-60 years and later declines [3,4]. Risk factors for seborrheic dermatitis include immunodeficiency, neurological diseases including Alzheimer and Parkinson disease, increased sebaceous gland activity, and exposures to drug treatment, including lithium, immunosuppressants, and dopamine antagonists [5]. Seborrheic dermatitis has no definitive cause; however, evidence suggests that pathogenesis begins with androgens and adrenal corticosteroids that stimulate sebaceous gland activity [4]. These hormones are modulated by obesity; therefore, nutrition and BMI may play a role in influencing the seborrheic dermatitis clinical course. Several studies suggest that nutrition can influence other inflammatory skin diseases, such as acne vulgaris, hidradenitis suppurativa, and psoriasis [6-8]. However, the magnitude of the effect on each disease may be small, and nutritional studies are inherently limited by recall bias. Typical treatment includes antifungals in combination with anti-inflammatories including topical corticosteroids and calcineurin inhibitors. However, long-term use of these corticosteroids can cause adverse effects, such as telangiectasia, and current treatments cannot eliminate this chronic disease. Therefore, other options like dietary modifications could assist with management and prevent recurrence [9]. Currently, there is no review evaluating the effects of nutrition and obesity on seborrheic dermatitis disease severity.

The goal of this review is to incorporate studies looking at diet, nutritional supplements, alcohol, obesity, and micronutrients in patients with seborrheic dermatitis into an organized framework that can be used by clinicians to make evidence-based recommendations and to provide useful insights into specific areas of further study. We aimed to answer the question of how diet, nutritional supplements, alcohol, obesity, and micronutrients affect seborrheic dermatitis prevalence, clinical course, severity, and subjective improvement in patients.

## Methods

The PRISMA (Preferred Reporting Items for Systematic Reviews and Meta-Analysis) statement was used to create this study. Case-control studies, cross-sectional studies, cohort studies, and randomized controlled trials with 5 or more subjects conducted on adult participants (>14 years) were included. Exclusion criteria were no case reports, case series, and review papers, as those did not provide a sufficiently high level of evidence. We also excluded studies that included any dietary or supplement intervention evaluated in the context of purposeful concurrent medication use. Eligible literature was any study evaluating BMI, waist circumference, micronutrients, alcohol use, or diet in relation to seborrheic dermatitis and any dietary or nutritional supplement intervention for seborrheic dermatitis. Eligible methodology to measure changes in seborrheic dermatitis severity included the SEborrheic Dermatitis Area and Severity Index (SEDASI) score, sebum levels, subjective seborrheic dermatitis severity, or seborrheic dermatitis severity evaluated by a physician.

We searched Scopus, PubMed, and MEDLINE (Ovid interface) for English language papers published between 1993 and 2023. The final search was conducted on April 16, 2023. The search terms consisted of (seborrheic dermatitis OR seborrheic eczema) AND (diet OR dietary patterns OR dietary activities OR nutrition OR supplements OR fruit OR vegetables OR gluten OR dairy OR sugars OR meat OR carbohydrates OR protein OR fats OR vitamin OR micronutrients OR minerals OR alcohol OR calorie OR weight loss OR weight changes OR obesity OR obesity reduction OR waist circumference OR body mass index OR BMI). Refer to Textbox 1 for full search strategy.

**Figure 1:**
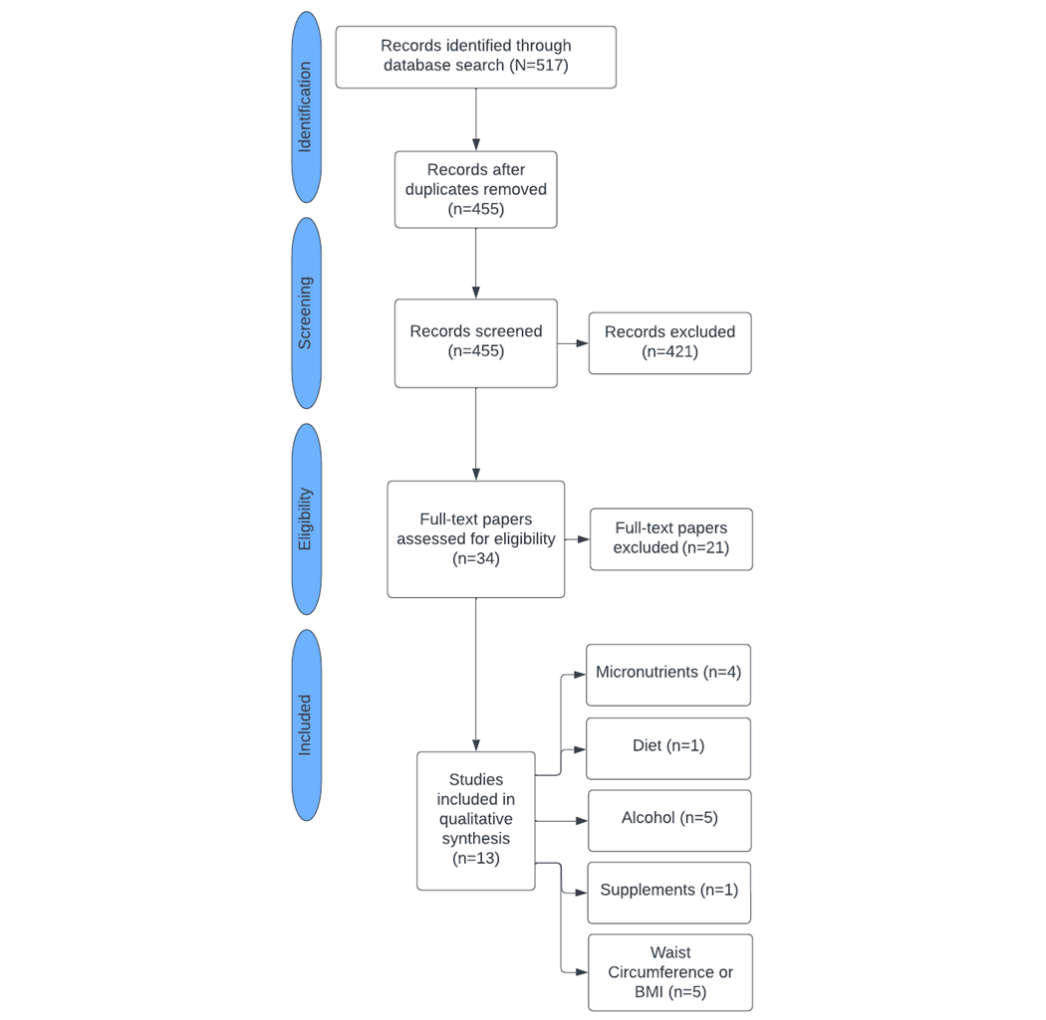
Study selection. All full-text papers were excluded due to a lack of inclusion criteria eligibility. A total of 3 studies included results that evaluated both BMI and alcohol use.

Literature search results were conducted by one person and exported to CADIMA to remove duplicates and review papers. This tool was used to ensure papers were uploaded to one place and so that reviewers could independently review included papers. In total, 455 unique studies were screened and assessed for eligibility by 2 reviewers working independently. Disagreements were resolved by a third reviewer’s decision. After applying inclusion and exclusion criteria, 13 studies (8 case-control, 3 cross-sectional, and 2 randomized control trials) involving 13,906 patients were selected for inclusion. Multiple studies included results that fit into more than 1 category, including 3 studies evaluating both BMI and alcohol use (Figure 1).

Table S1 in Multimedia Appendix 1 summarizes the included studies’ findings and evidence levels according to the ratings of the Oxford Centre for Evidence-based Medicine [10]. Levels of evidence are defined as (1) level 1, randomized trials or systematic reviews of randomized trials, cross-sectional studies, inception cohort studies, or nested case-control studies; (2) level 2, a systematic review of surveys, randomized trials, individual cross-sectional studies with consistent reference standards and blinding, inception cohort studies, or (exceptional) observational studies with dramatic effect; (3) level 3, Cohort studies, local nonrandom sample, nonconsecutive studies or studies without a consistently applied reference standard; (4) level 4, case series, case-control study, or historically controlled studies; and (5) level 5, mechanism-based reasoning. Level 1 represents evidence generally considered to be stronger, and level 5 represents evidence generally considered to be weaker. The Cochrane Collaboration’s tool for assessing the risk of bias was used to evaluate randomized control trials [11].

Although these tools intend to reduce bias in selected studies, bias can be transferred from the tools themselves. The Cochrane collaboration’s tool specifically assesses for the risk of bias, rather than for bias itself, and is more likely to miss bias associated with incomplete data and selective reporting [11]. Oxford-based Medicine levels of evidence help readers prioritize studies, but they should be used as a guide, rather than absolute, when determining the validity of a study [10].

**Search strategy for systematic review.**

- Database
  - Scopus, PubMed, and Ovid (MEDLINE interface)
- Search strategy
  - (seborrheic dermatitis OR seborrheic eczema) AND (diet OR dietary patterns OR dietary activities OR nutrition OR supplements OR fruit OR vegetables OR gluten OR dairy OR sugars OR meat OR carbohydrates OR protein OR fats OR vitamin OR micronutrients OR minerals OR alcohol OR calorie OR weight loss OR weight changes OR obesity OR obesity reduction OR waist circumference OR body mass index OR BMI)

## Results

### Micronutrients

A total of 3 studies evaluated micronutrient concentrations in patients with seborrheic dermatitis. Jahan et al [12] measured the levels of vitamins and minerals in patients with seborrheic dermatitis (n=75) compared with controls (n=76) in a case-control study, and concluded patients with seborrheic dermatitis had increased copper, manganese, iron, calcium, and magnesium concentrations (*P*<.001) and lower vitamin E concentrations (*P*=.009). Unfortunately, the study did not remeasure micronutrient concentrations after remission of seborrheic dermatitis, so it is not possible to conclude whether normalizing them is clinically beneficial. Another 10-week, randomized double-blind, placebo-controlled trial by Smith et al [13] (n=41) studied the impact of supplementation with weight-based, low-dose oral potassium bromide (3.5 mg/mL), sodium bromide (2.0 mg/mL), nickel sulfate (0.6 mg/mL), and sodium chloride (0.6 mg/mL) in a vehicle of purified water and 20% ethyl alcohol on seborrheic dermatitis severity measured by SEDASI. This study found an improvement in participants’ SEDASI score at 10 weeks of treatment (*P*=.03), with no significant difference in the frequency of adverse events between active and placebo groups [13].

Rahimi et al [14] evaluated serum levels of 25-hydroxyvitamin D (25(OH)D) in patients with seborrheic dermatitis (n=118) compared with healthy controls (n=171) in another case-control study [14]. They found that vitamin D deficiency was more prevalent in patients with seborrheic dermatitis than in controls (*P*=.01). This team neither obtained follow-up serum levels of vitamin D nor determined if subsequent supplementation resulted in seborrheic dermatitis improvement, thus it is difficult to conclude if supplementation is clinically beneficial. A separate case-control study evaluated serum zinc levels in patients with seborrheic dermatitis (n=43) compared with age and sex-matched, healthy controls (n=41) [15]. This study found lower serum zinc levels in patients with seborrheic dermatitis (*P*=.05); however, there was no correlation between serum zinc levels and seborrheic dermatitis duration or SEDASI score [15]. Although it evaluated the relationship between micronutrients and disease duration and severity, the study is limited by a small sample size and exclusion of severe patients with seborrheic dermatitis, limiting the generalizability of the data.

One study in the literature evaluates the association between seborrheic dermatitis and diet. This cross-sectional study by Sanders et al [16] (n=4379) examined if specific dietary patterns were associated with seborrheic dermatitis using participants of the Rotterdam Study (a prospective population-based cohort study of chronic diseases in the middle-aged and elderly population in the Netherlands) with a skin exam performed by a dermatology trained physician and a food frequency questionnaire with 389 questions evaluating the consumption of food over the past month. They found that the Western diet, characterized by meat, potato, and alcohol consumption, was associated with a higher risk for seborrheic dermatitis (adjusted odds ratio 1.34; *P*=.07) but only in female patients [16]. They also found that an increased amount of fruit in the diet was associated with a lower risk of seborrheic dermatitis (adjusted odds ratio 0.75; *P*=.03) [16]. Both associations compared the highest quartile of those most adherent to the dietary pattern with the lowest quartile of adherent participants [16]. Despite the popularity in recommending dietary changes, it is difficult to establish concrete conclusions about the viability of dietary manipulation as an adjunctive treatment due to limited data.

### Alcohol

Numerous studies found significant associations between regular alcohol use and an increased prevalence of seborrheic dermatitis [17-20] Furthermore, increased alcohol consumption is associated with a greater risk of seborrheic dermatitis flares (odds ratio 5.4; *P*=.08) [20]. Sharma et al [17] evaluated the quantity of alcohol consumed in a week, duration of alcohol intake, and seborrheic dermatitis duration in 196 males, who reported drinking ≥200 mL of pure alcohol weekly, that were referred for a dermatologic consult. They found an inverse relationship between seborrheic dermatitis prevalence and duration of alcohol intake, seborrheic dermatitis prevalence is inversely related to the duration of alcohol intake, meaning those with fewer years of alcohol use (*P*<.001) were more likely to have seborrheic dermatitis [17].

One cross-sectional study by Sanders et al [3] (n=5498) used Rotterdam study participants who underwent a full-body skin exam by dermatologists, and it compared patient characteristics for those with and without seborrheic dermatitis. They found no association between alcohol and seborrheic dermatitis; however, this study only included middle-aged and elderly patients, making it difficult to generalize to younger patients [3]. In addition, according to the findings by Sharma et al [17], seborrheic dermatitis would be more prevalent in younger patients who use alcohol, and this could account for the lack of association found by Sanders et al [3] when looking at an older population.

These studies are limited by their survey-based design, which inherently include self-reported alcohol intake, which may be unreliable. In addition, there are numerous uncontrolled, confounding factors, including smoking, tobacco use, and HIV, making it difficult to draw accurate conclusions about the effects of alcohol alone. Data related to alcohol and seborrheic dermatitis found in this review have weak and contradictory evidence, warranting further study.

### Supplements

Few nutritional supplements have been evaluated as an intervention for seborrheic dermatitis. One was evaluated in an 8-week, randomized, placebo-controlled trial by Zaeie et al [21] (n=80) was Triphala: a prebiotic. Patients with seborrheic dermatitis received 1 gram of Triphala twice a day for 8 weeks, then rated subjective symptomatic improvement from 1 to 100 [21]. Researchers assessed scalp sebum levels using a Sebumeter [21]. The Triphala group experienced both improvement in patient satisfaction (mean percentage of patients’ satisfaction was 37.91 in the Triphala group and 17.89 in the placebo group, *P*=.001) and scalp sebum levels (Triphala group: mean 103.67, SD 70.37; placebo group: mean 128.45, SD 73.90; *P*=.047) [21].

This study is limited by a small sample size, which prevents generalizability to broader populations and introduces doubt surrounding data reproducibility. More trials are necessary to elucidate if there is true efficacy of oral nutritional supplements on seborrheic dermatitis.

### Obesity

Some studies demonstrate no relationship between seborrheic dermatitis and BMI [10,19,20]; however, a case-control study by Akbaş et al [22] (n=101) compared patients with seborrheic dermatitis with age and sex-matched controls and found that seborrheic dermatitis was associated with higher BMI when compared with controls (*P*=.002). Savaş Erdoğan et al [23] (n=103) also evaluated the relationship between BMI, subjective seborrheic dermatitis severity, and SEDASI score in a case-control study with patients with seborrheic dermatitis and age, sex, and BMI-matched controls. Results showed a positive relationship between the SEDASI score and BMI (*r*=0.298; *P*=.03) but no relationship between subjective disease severity score and BMI (*P*=.62) [23]. Although there are studies to the contrary, most evidence indicates no relationship between BMI and seborrheic dermatitis severity [10,19,20,24]. Studies that suggest a causal relationship between the 2 are limited by numerous confounders, including ethnic background, socioeconomic status, and lifestyle factors.

Erdogan et al [23] (n=103) and Akbas et al [22] (n=101) conducted case-control studies comparing waist circumference in patients with seborrheic dermatitis to age, sex, and BMI-matched healthy controls. Both found that waist circumference was higher in the seborrheic dermatitis groups (*P*=.007 and *P*=.001, respectively). These studies are limited by small sample size [24].

## Discussion

The pathophysiology of seborrheic dermatitis is still not entirely understood; however, colonization of Malassezia, a fungus present on normal skin, is strongly associated with this condition [25]. Malassezia is found on sebum-rich skin and functions as a lipophilic yeast [25]. The metabolites of Malassezia induce inflammation, causing infiltration of natural killer cells and macrophages, and increased inflammatory cytokines such as interleukin 1α, 1β, and 6, and tumor necrosis factor α [26]. These inflammatory mediators can stimulate keratinocyte differentiation, resulting in dysfunction in the stratum corneum, disruption of the epidermal barrier, and perpetuation of an inflammatory response. This creates a cycle of skin barrier disruption that manifests the clinical features of seborrheic dermatitis [15,25].

Inflammation and oxidative stress are closely linked—oxidative stress causes inflammation, and inflammation precipitates oxidative stress [27]. Increased serum iron, copper, and manganese cause oxidative stress by catalyzing the creation of reactive oxygen species, which in turn leads to the development of inflammatory skin diseases like atopic dermatitis and psoriasis [28-30]. Systemic oxidative stress is also higher in patients with seborrheic dermatitis than in healthy subjects, suggesting a role in the pathogenesis of this disease [31]. Thus, the findings of Jahan et al [12] regarding elevated levels of serum iron, copper, and manganese in patients with seborrheic dermatitis may contribute to the cycle of oxidative stress, inflammation, and skin barrier disruption.

One study found dietary supplementation with low-dose oral potassium bromide, sodium bromide, nickel sulfate, and sodium chloride, reduced seborrheic dermatitis severity [13]. This compound consists of inorganic soluble mineral salts, but no evidence exists in the literature to explain this compound’s mechanism of action. More research is needed to determine the function these mineral salts play in modulating the epidermal barrier.

Zinc is an essential trace element that assists in cell growth, development, and differentiation and plays catalytic and structural roles in transcription factors, receptors, growth factors, cytokines, and enzymes [32]. It also possesses anti-inflammatory properties, including inhibiting polynuclear neutrophils chemotaxis and altering the production of interleukin-6 and tumor necrosis factor α, 2 proinflammatory cytokines produced by keratinocytes [33,34]. It also possesses antiandrogen activity by inhibiting 5 alpha-reductase type I expression [35]. These inflammatory and androgenic pathways are essential to the pathogenesis of seborrheic dermatitis, therefore lower serum zinc levels in patients with seborrheic dermatitis may represent a precipitating factor to disease development [15]. Importantly, there was no correlation between serum zinc levels and disease severity graded by SEDASI, so it may only be involved in the development of the disease rather than progression [15]. The authors postulated that this is due to the study’s small sample size and their inclusion of only mild seborrheic dermatitis. More studies are needed to identify if this relationship holds true for severe seborrheic dermatitis and if oral zinc supplementation is of clinical benefit.

Vitamin E is a fat-soluble vitamin and an important antioxidant that helps protect cell membranes from lipid peroxidation, minimizing oxidative damage [36]. Therefore, the low levels of vitamin E in patients with seborrheic dermatitis may contribute to an increased oxidative burden [12]. Supplementation with oral vitamin E showed early promising results in improving other inflammatory skin diseases, including atopic dermatitis and psoriasis [36]. Further research is needed to elucidate the role of vitamin E supplementation as an adjunctive therapy in seborrheic dermatitis.

Vitamin D plays a role in multiple skin processes, ranging from keratinocyte proliferation, differentiation, and apoptosis to immunoregulatory processes and barrier maintenance [37]. Vitamin D enhances the synthesis of structural proteins and mediates immunosuppressive action in the skin [37]. Thus, the lower 25-hydroxyvitamin D levels in patients with seborrheic dermatitis found by Rahimi et al [14] and Borda and Wikramanayake [25] may decrease these protective functions, generating epidermal barrier dysfunction in seborrheic dermatitis. Vitamin D deficiency also plays a role in other inflammatory skin pathologies, such as psoriasis and atopic dermatitis; supplementation of vitamin D3 and vitamin D analogs is effective against psoriasis [37]. Further research is needed to determine the significance of vitamin D deficiency in seborrheic dermatitis and the efficacy of interventional supplementation.

Prebiotics and probiotics possess antimicrobial properties and play a role in the inflammatory response and skin barrier function [38]. Probiotics are live microorganisms while prebiotics are nondigestible carbohydrates that induce the growth of probiotic bacteria [38]. Pre- and probiotics are beneficial for several dermatologic conditions, including dandruff and seborrhea, but their use in seborrheic dermatitis is limited [39]. Triphala, a polyphenol-rich prebiotic, is one of the few dietary supplements that has been tested as a seborrheic dermatitis treatment [21]. It has antioxidant properties and acts as a skin protectant for human skin cells in vitro [40]. The study was limited by a small sample size, but Triphala is a potential adjunctive treatment for seborrheic dermatitis, but more extensive clinical trials are necessary to fully understand its efficacy and safety.

Through assessment with the Cochrane tool for assessing risk bias, a tool validated for randomized controlled trials, some biases were observed [11]. Authors denoted a small sample size as a limitation that could inherently give the trial attrition bias. Of the 81 patients originally starting the trial, 80 completed it as participants could abandon it at any time. Patients were blinded due to placebo intervention being used in half of the group. Assessors were blinded to the fact whether the subject received the active treatment or the placebo. The authors stated that the use of a Sebumeter made the data objective, which could have led to some detection bias to overestimate the validity of the results. In addition, performance bias could be at risk due to overconfidence in blinding of the capsules. Researchers claimed that capsules were matched to size, color, and consistency [21].

The Western diet includes a high volume of meat, potato, and alcohol, and a low volume of foods rich in fiber, vitamins, and minerals; its popularity has grown substantially over the past few decades. Long-term consumption of foods popular in the Western diet can negatively impact health by promoting weight gain, activating the immune system, and causing pathological changes in lipids and metabolism [41]. Only 1 study evaluates adherence to specific dietary patterns and their association with seborrheic dermatitis. This study found that patients with higher fruit intake had a decreased likelihood of seborrheic dermatitis [16]. These results align with research on other inflammatory skin diseases, including eczema incidence, which is negatively associated with increased fruit intake [42]. Fruits contain high levels of vitamins and flavonoids, which reduce inflammation and may modulate the inflammatory response in seborrheic dermatitis that contributes to skin barrier dysfunction [42].

This same study found that higher adherence to the Western diet in females was associated with an increased prevalence of seborrheic dermatitis, possibly due to increased chronic inflammation associated with the diet [16,43]. This association was not present in males, which may be explained by known differences in dietary response between the sexes [44,45].

It should be further highlighted that the use of diet to mitigate disease severity is popular in the mainstream, but existing literature suggests a tenuous relationship. A 2015 study assessed the validity of memory-based dietary assessment methods on informing dietary policies and found that they are inherently flawed, as they necessarily involve subjective memory recall with no objective data [46]. Therefore, retrospective studies of food consumption are prone to bias and are generally limited.

Chronic alcohol use is linked to a variety of skin conditions and the earliest clinical manifestations of alcohol use disorder are cutaneous [47]. There is a known association between regular alcohol use and seborrheic dermatitis, likely resulting from immunosuppression, malnutrition, poor hygiene, vitamin B deficiency, and other confounders [17-20]. One study did not find an association between regular alcohol use and seborrheic dermatitis adjusted for possible confounders, including demographic, socioeconomic, and medical information, calling into doubt conclusions drawn from unadjusted studies [10]. A separate crossover study found that more recent consumption of alcohol was associated with seborrheic dermatitis flares; however, this also correlated with increased reported stress levels, introducing an additional confounder [20]. Given the conflicting nature of these data, it is unclear whether alcohol is an independent determinant of seborrheic dermatitis clinical course, thus controlled interventional studies are necessary.

Current literature on the association between BMI and seborrheic dermatitis is mixed, although larger studies indicate no association [10,19,20,22,23]. The conflicting nature of these data may reflect BMI’s poor predictive value in judging metabolic health, which is associated with seborrheic dermatitis [22,48,49].

An arguably more accurate indicator of obesity is waist circumference, with multiple case-control studies showing waist circumference significantly higher in patients with seborrheic dermatitis compared with controls [22-24]. Numerous inflammatory markers are higher in those with obesity, which may contribute to initiating or aggravating seborrheic dermatitis [46]. Abdominal obesity can also lead to dyslipidemia, another factor associated with seborrheic dermatitis [22,23,48].

### Conclusions

More studies are needed to determine how micronutrients, diet, supplements, and obesity affect seborrheic dermatitis. This review sheds light on promising areas of research that require further study but highlights that current data are limited. Low levels of serum zinc, vitamin D, and vitamin E in patients with seborrheic dermatitis suggest a role for interventional studies evaluating the benefits of supplementation. The prebiotic Triphala may also improve seborrheic dermatitis; however, larger studies with more severe seborrheic dermatitis are needed to evaluate its true potential.

The negative consequences of a Western diet, alcohol use, obesity, and the benefits of fruit consumption are well known; however, to fully understand their specific relationships to seborrheic dermatitis, further cohort or interventional studies are needed. As it stands, information on diet-based therapy for seborrheic dermatitis is conflicting and limited, thus future studies are warranted.

## Appendix

Multimedia Appendix 1 Summary of included studies.

Multimedia Appendix 2 Preferred Reporting Items for Systematic Reviews and Meta-Analysis (PRISMA) Checklist.

## Abbreviations

SEDASI: SEborrheic Dermatitis Area and Severity Index.
PRISMA: Preferred Reporting Items for Systematic Reviews and Meta-Analysis

## Data Availability

The data sets generated during and/or analyzed during this study are available from the corresponding author on reasonable request.
