# Supplementary material for: Nutrition, Obesity, and Seborrheic Dermatitis: Systematic Review
Source: JMIR Dermatol. 2024 Aug 5;7:e50143. doi: 10.2196/50143 (PMC11333864; doi:10.2196/50143)
Supplement: Multimedia Appendix 1 [file derma_v7i1e50143_app1.docx]

**Table S1**

| Study | | Study design | Participants | Sample, n | Intervention | Findings | Limitations | Evidence level [10] |
| --- | --- | --- | --- | --- | --- | --- | --- | --- |
| **Micronutrients** | | | | | | | | |
|  | Jahan et al (2021) [12] | Case-control study | - Dermatologist diagnosed patients with SD^a^ - Controls: age or sex-matched healthy patients | 151 | Evaluate serum trace elements, micronutrients, antioxidants, malondialdehyde, and immunoglobulins in patients with SD. | Mean ± SEM^b^ for   - Serum zinc (mg/L) patients with SD: 1.430 ± 0.11, HCs^c^: 1.308 ± 0.09; *P*=.289 - Serum copper (mg/L) patients with SD: 2.136 ± 0.10, HCs: 0.950 ± 0.05; *P*<.001 - Serum manganese (mg/L) patients with SD: 15.830 ± 0.85 HCs: 7.022 ± 0.46; *P*<.001 - Serum iron (mg/L) patients with SD: 2.248 ± 0.14 HCs: 1.130 ± 0.11; *P*<.001 - Serum calcium (mg/L) patients with SD: 185.040 ± 5.89 HCs: 99.580 ± 1.77; *P*=.001 - Serum magnesium (mg/L) patients with SD: 29.640 ± 0.55 HCs: 20.640 ± 0.27, *P*<.001 - Serum vitamin A (μmol/L) patients with SD: 0.403 ± 0.07 HCs: 0.374 ± 0.03, *P*=.707 - Serum vitamin E (μmol/L) patients with SD patients: 5.564 ± 0.39 HCs: 7.074 ± 0.37, *P*=.009 | Did not measure these parameters after the remission of SD.  This study did not receive any funding. | 4 |
|  | Rahimi et al (2021) [14] | Case control study | Patients aged 18-65 years referred to the general outpatient clinic of Imam Sajjad Hospital and seen by a single dermatologist  Controls: healthy adults attending clinic for cosmetic consultations | 289 | Determine serum level of 25(OH)D^d^ in patients with clinically manifested facial or scalp SD | - 25(OH)D was significantly lower in the case group compared with the control group (20.71 ± 8.16 vs 23.91 ± 7.78; *P*=.007) - Vitamin D insufficiency: more prevalent among healthy individuals - Vitamin D deficiency: more prevalent in cases (difference was statistically significant among groups, *P*=.013) | Lack of follow-up to see if supplementation of vitamin D improved SD  This study received funding from Mazandaran University for Medical Sciences Vice Chancellor of Research. | 4 |
|  | Karabay and Cerman (2019) [15] | Case control study | Patients diagnosed with SD by clinical or histopathological examination from dermatology outpatient clinic  Controls: healthy age or sex-matched patients with no evidence of SD from hospital staff volunteers | 84 | Determine the association between SD and serum zinc levels  SD was graded according to SEDASI^e^ | - Significantly lower serum zinc levels in SD patient’s vs control group (79.16±12.17 vs 84.88±13.59, *P*=.045) - No correlation found between serum zinc levels and disease duration (*P*=.658) or SEDASI scores (*P*=.273) | Small sample size  Only patients with mild or moderate SD included  This study did not declare the funding source | 4 |
|  | Smith et al (2002) [13] | Double-blind placebo-controlled study | Patients 20-77 years with dermatologist diagnosed SD and a minimum of 20% affected scalp, face, or combined surface area  Controls: age or sex-matched patients with diagnosed SD | 41 | Evaluate therapeutic potential of low-dose oral administration of weight-based dosage (<100 pounds=2.0 mL/day, 100-200 pounds = 4.0 mL/day, >200 pounds=8.0 mL/day) of potassium bromide (3.5 mg/mL), sodium bromide (2.0 mg/mL), nickel sulfate (0.6 mg/mL) and sodium chloride (0.6 mg/mL) on SD severity over 10 weeks  SD severity measured by SEDASI | - Percent improvement in SEDASI at 10 weeks: Placebo=-10.82%   Active=38.5%  (*P*=.0302)   - Significant improvement in SD due to active treatment after crossover did not occur until 10 weeks of dosage (*P*=.0035) - Three patients lost to dropout due to intervention side effects (placebo: nausea, flu-like symptoms; active: stomach problems) but no significant difference in frequency of side effects for active vs placebo (*P*=.80) | Small sample size  This study did not declare funding source | 2 |
| **Diet** | | | | | | | | |
|  | Sanders et al (2019) [16] | Cross-sectional study | Rotterdam study participants who underwent FBSE^f^ by a dermatologist and had complete nutrition data  Controls: participants without SD | 4379 | Evaluate whether total dietary antioxidant intake (using FRAP^g^ score) or specific defined dietary patterns (using an FFQ) were associated with SD | - No association between antioxidant intake and SD (quartile 1 vs quartile 4: adjusted odds ratio=0.94, 95% CI =0.73–1.19; *P*=.88) - Western diet: associated with a higher risk for SD (quartile 1 vs quartile 4: adjusted odds ratio=1.34, 95% CI=1.03–1.75; *P*=.07) - Stratification of Western diet and sex:  Males: no significant association between Western pattern and SD Females: higher adherence to Western pattern was associated with an increased risk of SD - Adherence to the fruit pattern: associated with a lower risk for SD (quartile 1 vs quartile 4: adjusted odds ratio=0.75, 95% CI=0.58–0.97; *P*=.03) - Vegetable or fat patterns: no association with the presence of SD | Cross-sectional design does not allow for causal inferences   The study covers only the middle-aged and elderly population thus generalizability to younger patients is limited  The disease severity and distribution of SD were not specifically documented during the FBSE so no relationship between the location or severity of the disease and the associations with the diet components can be determined  The use of an FFQ^h^ to assess dietary intake is prone to measurement error  This study was funded by Erasmus Medical Center and Erasmus University Rotterdam; Netherlands Organization for the Health Research and Development (ZonMw); the Research Institute for Diseases in the Elderly (RIDE); the Ministry of Education, Culture and Science; the Ministry for Health, Welfare and Sports; the European Commission (DG XII); and the Municipality of Rotterdam | 3 |
| **Alcohol** | | | | | | | | |
|  | Sharma et al (2017) [17] | Case-control study | Subjects fulfilling criteria of Royal College of Psychiatry for chronic drinking (>200 ml of pure alcohol or week)  Controls: age or sex-matched patients in the dermatology outpatient department | 196 | Correlate the spectrum of dermatoses in chronic alcoholics with the quantum or duration of alcohol intake and raised liver transaminases | - Prevalence of SD in cases vs controls: 11.2% vs 1% (OR^i^: 12.26; *P*<.001) - SD was inversely related to duration of alcohol intake (12±6.094 vs 21.02±9.179 years, *P*<.001), ie, more in those with lesser years of intake and of lesser age (35.68±5.093 vs 44.26±8.852 years; *P*<.001) | Small sample size  Nonreliable history of alcohol intake  Inability to eliminate the confounding bias due to factors such as smoking, tobacco, and HIV infection | 4 |
|  | Rao (2004) [18] | Case-control study | Patients attending alcohol deaddiction camps underwent detailed skin exam  Controls: age or sex-matched nonalcoholics | 200 | Determine cutaneous changes in chronic alcoholics | - Prevalence of SD in cases vs controls: 11.5% vs 2.5% (*z* test=3.6; *P*<.001) | Small sample size  No standardized history of alcohol intake  Inability to eliminate the confounding bias due to factors such as smoking, tobacco, and HIV infection  This study did not use funding. | 4 |
| **Supplements** | | | | | | | | |
|  | Zareie et al (2022) [21] | RCT^j^ | Participants with scalp seborrhea aged 14–50 years were randomized to Triphala or placebo groups | 80 | 1 g of Triphala (standardized as 91.82 ± 0.5 mg gallic acid) or placebo (wheat flour) was administered BID^k^ for 8 weeks  Scalp sebum levels were detected using Sebumeter SM 815  Treatment satisfaction was measured using a score between 0 and 100 | - Triphala group: experienced 25.34 scores (95% CI, 0.39–50.29; *P*=.047) more improvement in scalp sebum levels compared with the placebo group - Mean percentage of patients’ satisfaction: (*P*=.001) 37.91 in the Triphala group 17.89 in the placebo group | Small sample size  This study did not declare funding source | 1 |
| **Obesity or BMI** | | | | | | | | |
|  | Erdogan et al (2022) [23] | Case-control study | Participants were >18 years old and diagnosed with SD  Controls: age or sex or BMI^l^ matched healthy patients | 103 | Evaluate MS^m^ and glucose metabolism disorders in patients with SD graded by SEDASI score and subjective disease severity score | - Waist circumference (*P*=.007): Significantly higher in the SD group compared with the control group - Significantly positive relationship between the SEDASI score and BMI (*r*=0.298, *P*=.030) - No relationship between subjective disease severity score and BMI (*P*=.62) | Small sample size  This study did not declare funding source | 4 |
|  | Akbas et al (2022) [22] | Case-control study | Participants were >18 years old and without known diabetes mellitus, hypertension, and coronary artery disease who were clinically diagnosed with SD in clinic  Controls: healthy individuals who came to clinic for general care | 101 | Investigate the relationship between the presence of MS parameters, SD severity graded by SEDASI score and duration of disease | - Patients with SD: Mean BMI was 26.9 (standard deviation: 7.2)  Patients with control: Mean BMI was 23.5 (standard deviation: 4.1) - BMI of SD patients was significantly higher compared with the controls (*P*=.002) - Waist circumference of SD patients was significantly higher than the control group (*P*=.001) | Small sample size  This study did not receive funding | 4 |
|  | Sanders et al (2018) [3] | Cross-sectional study | Rotterdam study participants who underwent an FBSE by a dermatologist | 5498 | Evaluate lifestyle and physiological determinants associated with SD | - No association between BMI and SD (Adjusted OR for BMI (95% CI): 25-30=1.05 (0.87-1.27), >30=1.12 (0.90-1.39)) - No association between alcohol and SD (Adjusted OR for alcohol consumption (95% CI)=1.01 (0.99-1.01) | Cross-sectional design does not allow for causal inferences  The study covers only the middle-aged and elderly population thus generalizability to younger patients is limited  This study is funded by Unilever. The parent Rotterdam Student is funded by Erasmus Medical Center, Erasmus University Rotterdam, Netherlands Organization for Health Research and Development, the Research Institute for Diseases in the Elderly, the Ministry of Education, Culture and Science, the Ministry for Health, Welfare and Sports the European Commission, and the Municipality of Rotterdam | 3 |
|  | Bas et al (2016) [19] | Cross-sectional study | Family Medicine patients >20 years of age that reflected the population demographics of Tokat Province | 2325 | Determine the prevalence and frequently encountered risk factors associated with SD and Psoriasis | - BMI and SD: No significant association - Alcohol use and SD: Significant difference between participants (*P*=.011) | Cross-sectional design does not allow for causal inferences  This study did not declare funding source | 3 |
|  | Lancar et al (2020) [20] | Case-control and crossover study | Case-control study: patients with either a past history of or an active form of SD   Control patients: age or sex-matched outpatients seen during the same period  Cross Over Study: patients who visited the same dermatologist for a second time and had presented with active SD during the first visit and inactive SD during the second visit or vice versa | Case-control study: 378  Crossover study: 81 | Evaluate risk factors for SD flares in a population of French patients consulting the same office-based dermatologist | - BMI and SD: No significant relationship (*P*=.52) - Regular alcohol consumption and SD: OR=10.2, 95% CI = 2.0-52.6, *P*=.01 - Risk of SD flares and higher consumption of alcohol: OR=5.4, CI=0.8–34.9, *P*=.08 | Confounding due to lack of controls for ethnic background, socioeconomic status, and lifestyle factors  This study received funding from pharmaceutical companies but did not specify the amount. | 2 |

^a^SD: seborrheic dermatitis.

^b^SEM: standard error of the mean.

^c^HC: healthy controls.

^d^25(OH)D: 25-hydroxyvitamin D.

^e^SEDASI: SEborrheic Dermatitis Area and Severity Index.

^f^FBSE: full body skin exam.

^g^FRAP: ferric-reducing ability of plasma.

^h^FFQ: food frequency questionnaire.

^i^OR: odds ratio.

^j^RCT: randomized controlled trial.

^k^BID: twice daily.

^l^BMI: body mass index.

^m^MS: metabolic syndrome.
